# En Réponse Au Projet De Loi 2 : Associations Entre Les Démarches Légales D’affirmation Du Genre et Deux Indicateurs De Bien-être Chez Des Personnes Trans et Non-Binaires Du Québec

**DOI:** 10.1177/07067437221090088

**Published:** 2022-03-31

**Authors:** Julie Christine Cotton, Alexa Martin-Storey, Yann Le Corff, Séré Gabriel Beauchesne Lévesque, Annie Pullen Sansfaçon

**Affiliations:** 1 Département des sciences de la santé communautaire, Faculté de médecine et des sciences de la santé, 7321 Université de Sherbrooke, Sherbrooke, QC, Canada; 2 Département de Psychoéducation, Faculté d’éducation, 7321 Université de Sherbrooke, Sherbrooke, QC, Canada; 3 Département d’orientation professionnelle, Faculté d'éducation, 7321 Université de Sherbrooke, Sherbrooke, QC, Canada; 4 Laboratoire inclusif de recherche et développement, Université de Sherbrooke, Coordonnateur chez TransEstrie, Sherbrooke, QC, Canada; 5 École de travail social, Faculté des arts et des sciences, 5622 Université de Montréal, Sherbrooke, QC, Canada

**Keywords:** well-being, psychological distress, life satisfaction, legal transition, gender identity, transgender, non-binary

## Abstract

**Mots-clés** bien-être, détresse psychologique, satisfaction devie, transition légale, identité de genre, trans, non-binaire

## Introduction

Chez les personnes trans et non-binaires (TNB), le fait d’avoir pu compléter une transition souhaitée est associé à une meilleure satisfaction de vie chez ces dernières, ainsi qu’à moins de détresse psychologique, comparativement aux personnes qui n’entament pas de transition.^1^ Si nous connaissons davantage les bienfaits de transitionner en général^2^ et les impacts de démarches spécifiques sur le bien-être, comme les transitions médicales,^3^ nous en savons encore peu sur les impacts des démarches de transition légales.^4^

Dans la foulée des débats sociojuridiques actuels au Québec, depuis l’annonce du controversé projet de loi 2,^5^ l'objectif de cette étude est d'examiner les associations entre le fait d’entreprendre une démarche de transition légale ou administrative chez les personnes TNB et deux indicateurs de leur bien-être, en tenant compte de l’âge, de l'éducation, du sexe assigné à la naissance, du statut racial et de l'identité de genre de ces personnes. Nous posons l’hypothèse que le fait d'avoir entrepris au moins une démarche de ce type sera associé à des niveaux moindres de détresse psychologique et à des niveaux plus élevés de satisfaction de vie.

## Échantillon et Procédures

Cette recherche s’inscrit dans le cadre d’une enquête en ligne menée en 2018 sur l’accès aux services, les besoins et les enjeux psychosociaux des personnes TNB. Le questionnaire de l’enquête, développé en collaboration avec des personnes des communautés TNB, est disponible auprès de la première auteure. Un total de 240 personnes québécoises âgées entre 14 et 68 ans (M = 27.97, ÉT = 11.61) a consenti à la recherche. De ce nombre, 157 ont répondu aux questions sur les démarches légales ou administratives d’affirmation du genre et ont été retenues pour les analyses. Aucune différence n’a été observée sur les variables à l’étude entre les personnes retenues pour les analyses et celles ayant été exclues.

## Méthode

La détresse psychologique a été mesurée à l’aide de la *Kessler Psychological Distress Scale* (K6) (Kessler *et al*., 2010). La satisfaction de vie a été évaluée à l’aide de la *Satisfaction with Life Scale* (Blais, Vallerand, Pelletier & Brière, 1989). Les démarches légales ou administratives impliquaient le fait d’avoir entrepris au moins une de ces démarches : (1) changement du nom auprès de la direction de l'état civil, (2) changement de la mention du sexe auprès de la direction de l'état civil, (3) changements administratifs auprès d'autre(s) organisme(s), (4) Autre(s). Cette variable a été dichotomisée (0 = aucune démarche, 1 = au moins une démarche). Concernant les covariables, les personnes répondantes ont été questionnées sur leur âge, leur niveau d'éducation, leur identité de genre et leur sexe assigné à la naissance (0 = féminin et 1 = masculin), car seules ces deux options ont été sélectionnées.

## Stratégie D'analyse

Des analyses de régression par la méthode des moindres carrés ordinaires a été conduite avec MPlus 7.4, avec pour variable dépendante la détresse psychologique dans un premier modèle et la satisfaction de vie dans un second modèle. Le fait d’avoir entrepris ou non au moins une démarche de transition légale a été utilisé comme variable indépendante dans les deux modèles, en contrôlant l’âge, l’appartenance ethnoculturelle, le niveau d’éducation, le sexe assigné à la naissance et l’identité de genre actuelle. L’estimateur MLR a été utilisé puisque certaines variables avaient une distribution asymétrique ou aplatie. Les données manquantes dans l’échantillon à l’étude se sont révélées entièrement aléatoires (Little's MCAR χ^2^ (8) = 4.80, *p* = 0.78). Au total, 8% des données étaient manquantes mais une suppression par liste aurait conduit à une attrition de 41% de l’échantillon, avec un effet délétère sur la puissance statistique. La méthode d’imputation *Full information maximum likelihood* a donc été utilisée pour gérer les données manquantes.

## Résultats

Tel qu’illustré au tableau 1, compte tenu des variables de contrôle, le fait d'avoir entrepris au moins une démarche de transition légale ou administrative est associé à un niveau significativement moindre de détresse psychologique chez les personnes répondantes. Cette diminution est de 37% d'un écart-type de la détresse (environ 2 points). Le fait d'avoir entrepris au moins une démarche de transition légale ou administrative est associé à une satisfaction de vie significativement plus élevée, avec une augmentation de 38% d’un écart-type de la satisfaction de vie (environ 2.5 points).

**Tableau I.** Statistiques Descriptives et Régressions Multiples Pour la Détresse Psychologique et la Satisfaction de vie (N = 157).

	Statistiques descriptives			Détresse psychologique				Satisfaction de vie			
				M = 11.32, ÉT = 5.38				M = 20.77, ÉT = 6.85			
				Min = 0.00, Max = 24.00				Min = 5.00, Max = 35.00			
	M(%)	ÉT	Min	Max	B		Beta		B		Beta
Au moins une démarche de transition légale ou administrative	50.3%			−1.99		−.19*		2.58		0.19*	
Âge	28.38	12.18	14.00	68.00	−0,14		−.31**		0.10		0.18*
Statut racial (référent : Blanc comme référence)	10.4%			1.77		,10		−3.39		−0.15	
Éducation (référent : école secondaire uniquement)	70.4%			−0.34		-,03		2.53		0.17	
Sexe assigné à la naissance (référent : garçon)	36.0%			−0.45		-,04		−1.32		−0.09	
Identité de genre (référent : non-binaire)	17.9%			−1.12		-,08		−0.51		−0.03	
R^2^				0.17**				0.15**			

* = *p* < 0.05; ** = *p* < 0.01. M = moyenne; ÉT = Écarte type; Min = Minimum; Max = Maximum.

## Discussion

Nos résultats vont dans le même sens qu’une récente étude démontrant les bienfaits de l'utilisation du nom choisi sur la santé mentale des jeunes TNB^6^. Les démarches de transition légales et administratives permettent de légitimer le nom choisi dans différentes sphères de vie des personnes TNB, en plus de permettre la reconnaissance juridique et formelle de leur identité de genre. Nos données suggèrent que le fait de faciliter l’accès aux démarches de transition légales et d’adapter les qualités de l’état civil (mentions de sexes, titres parentaux) pour qu’elles reflètent adéquatement la pluralité des genres pourrait affecter positivement la santé des populations TNB au Québec et ailleurs.
